# Socio-economic and ethnic disparities in childhood cancer survival, Yorkshire, UK

**DOI:** 10.1038/s41416-023-02209-x

**Published:** 2023-02-24

**Authors:** K. J. Cromie, N. F. Hughes, S. Milner, P. Crump, J. Grinfeld, A. Jenkins, P. D. Norman, S. V. Picton, C. A. Stiller, D. Yeomanson, A. W. Glaser, R. G. Feltbower

**Affiliations:** 1 Leeds Institute for Data Analytics, School of Medicine, University of Leeds, Leeds, UK; 2 Leeds Institute of Medical Research, School of Medicine, University of Leeds, Leeds, UK; 3 Leeds Teaching Hospitals NHS Trust, Beckett Street, Leeds, UK; 4 Sheffield Children’s NHS Foundation Trust, Clarkson Street, Sheffield, UK; 5 School of Geography, University of Leeds, Leeds, UK; 6 National Disease Registration Service, Public Health England, London, UK

**Keywords:** Cancer epidemiology, Paediatric cancer

## Abstract

**Background:**

Establishing the existence of health inequalities remains a high research and policy agenda item in the United Kingdom. We describe ethnic and socio-economic differences in paediatric cancer survival, focusing specifically on the extent to which disparities have changed over a 20-year period.

**Methods:**

Cancer registration data for 2674 children (0–14 years) in Yorkshire were analysed. Five-year survival estimates by ethnic group (south Asian/non-south Asian) and Townsend deprivation fifths (I–V) were compared over time (1997–2016) for leukaemia, lymphoma, central nervous system (CNS) and other solid tumours. Hazard ratios (HR: 95% CI) from adjusted Cox models quantified the joint effect of ethnicity and deprivation on mortality risk over time, framed through causal interpretation of the deprivation coefficient.

**Results:**

Increasing deprivation was associated with significantly higher risk of death for children with leukaemia (1.11 (1.03–1.20)) and all cancers between 1997 and 2001. While we observed a trend towards reducing differences in survival over time in this group, a contrasting trend was observed for CNS tumours whereby sizeable variation in outcome remained for cases diagnosed until 2012. South Asian children with lymphoma had a 15% reduced chance of surviving at least 5 years compared to non-south Asian, across the study period.

**Discussion:**

Even in the United Kingdom, with a universally accessible healthcare system, socio-economic and ethnic disparities in childhood cancer survival exist. Findings should inform where resources should be directed to provide all children with an equitable survival outcome following a cancer diagnosis.

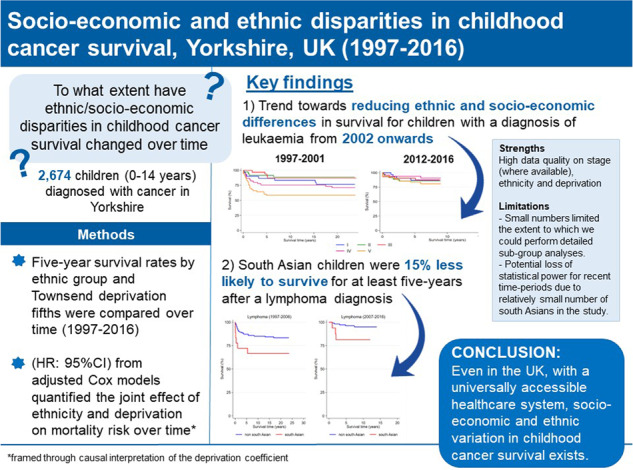

## Introduction

Recent studies based on both regional and national cancer registrations have shown improvements in survival rates for children diagnosed with cancer in the United Kingdom (UK), with survival estimates consistently exceeding 84% for children diagnosed before their 15th birthday [1, 2]. However, survival statistics for broad tumour groups reflect a heterogeneous mix of cases [3], and not all will have benefitted equally from these advances.

Social gradients in outcomes exist across a range of child health conditions, such as asthma, mental illness and adiposity [4–9]. Socio-economic inequalities in cancer survival have been extensively described for many adult malignancies, with individuals from more socio-economically deprived areas having poorer outcomes compared to those from more affluent backgrounds [10, 11]. A similar trend has been observed for children with paediatric leukaemia in the UK [12, 13]: children from more socio-economically deprived backgrounds diagnosed with acute lymphoblastic leukaemia (ALL) prior to 2010 have been found to be at an 86% increased risk of death compared to children from less deprived areas [13].

A small number of UK studies [14–17] have described differences in survival outcome by ethnic group. Findings include a 25% increased relative risk of death for children of south Asian ethnic origin diagnosed with ALL between 1981 and 1996 compared to White children [14], and a twofold increased risk of death for south Asian children (aged 14 and under) diagnosed with lymphoma up until 2005 compared to non-south Asians [15].

There is growing evidence to suggest that ethnicity and socio-economic status are important determinants of long-term paediatric cancer survival outcomes in the UK [12–17]. However, to date, most studies documenting the joint or independent effect of socio-economic status and/or ethnicity on childhood cancer survival [12–16, 18] have not examined temporal changes. Systematic national estimates of socio-economic gradients or patterns of ethnic inequalities in childhood cancer survival over extended periods of time are not available [8]. Consequently, little is known about how the population-level inequalities impacting on outcomes have changed over time for childhood cancer populations, if at all.

The Yorkshire and the Humber region in the UK has a total population of 5.5 million, 2 million of whom are aged under 30 years [19]. Spanning an area of 15,000 square kilometres, the region comprises a diverse mix of urban and rural communities, with a significant ethnic minority population that is predominantly of south Asian origin (comprising 6% of the Yorkshire & the Humber population in the 2011 Census [20]) and mainly resident in parts of West Yorkshire. An estimated 60% of the south Asian population in Yorkshire originates from Mirpur in rural Pakistan [21]. This makes Yorkshire one of the few regions in the UK that allows for detailed analysis of a relatively homogeneous, second and third generation south Asian population.

Establishing the existence of and determining the reasons for childhood health inequalities remains a research and government priority for the UK [8, 9]. This study aimed to utilise high-quality population-based data on children (aged 0–14 years) diagnosed with cancer between 1997 and 2016, in combination with a validated method of ethnicity assignment using linked hospital episode statistics data [22], to explore patterns of socio-economic and ethnic variation in survival outcomes over time in a regional paediatric cancer population in the UK.

## Materials and methods

### Patients

Cases were extracted from the Yorkshire Specialist Register of Cancer in Children and Young people (YSRCCYP), a regional population-based database containing detailed demographic, diagnostic and clinical information on all children and young adults diagnosed with cancer since 1974. Data from 2674 children aged 0–14 years, resident in the Yorkshire & Humber region, UK who were diagnosed with a malignancy or a borderline/benign central nervous system (CNS) tumour between 1997 and 2016 were included, based on allocation of a recognised International Childhood Cancer Classification (ICCC) code. Any individuals who had a primary cancer diagnosis prior to 1997 or subsequent cancer diagnosis post 31 December 2016 were not included. The age range of 0–14 years was selected to facilitate direct comparison of childhood cancer survival outcomes in Yorkshire with those presented in previously published UK studies, during overlapping time periods [12, 13, 15].

Diagnoses were categorised according to the ICCC, third edition (ICCC-3) [23]. To retain statistical power, diagnoses were grouped into four main categories: leukaemia, lymphoma, central nervous system (CNS) tumours, and other solid cancers, corresponding to ICCC codes I, II, III and IV–XII, respectively. All patients were proactively followed-up every 2 years to ascertain their vital status with minimal loss to follow-up (<0.1%). Cases ascertained by death certificate data only were discounted (*n* = 14). For estimation of survival rates for individuals with multiple primary tumours (*n* = 40), only the first cancer for each patient was retained in the analysis. Each registered case was censored for follow-up on 31 December 2020 or if appropriate at the time of earlier death, resulting in all cases having a potential follow-up period of at least 4 years.

White blood cell count was used as a proxy for stage when modelling leukaemia survival rates; lymphoma stage was assessed using the Ann Arbor staging system; CNS tumours were categorised according to World Health Organisation grade (low grade I–II/high grade III–VI). Staging for other solid tumours was based on The TNM Classification of Malignant Tumours.

Assignment of ethnic group (south Asian (Pakistani, Indian, or Bangladeshi origin) and non-south Asian (White, or ‘Other’ ethnicities comprising Black, Chinese and other Asian children) was based primarily on linked inpatient hospital episode statistics data [22], which records ethnic group based on 1991 and 2001 Census categories (Table S2). Where this information was missing (*n* = 174), results from *Onomap* naming algorithms were used, in line with previous approaches [15, 24]. Combining ethnicity data from multiple sources ensured complete and accurate ethnicity data was available for all individuals. Treating physicians from the two Principal Treatment Centres (PTCs) in the Yorkshire region (Leeds Teaching Hospitals Trust and Sheffield Children’s Hospital) were contacted to independently validate ethnicity for a random subset of patients (*n* = 40). Ethnicity was assigned as either south Asian (*n* = 233) or non-south Asian (*n* = 2,434) for calculation of 5-year survival rates due to small within-group numbers and to facilitate comparison of survival trends with other published studies [15].

Townsend deprivation index was used as a measure of area-based material deprivation for each individual [25]. The index is based on official statistics on rates of unemployment, non-home ownership, household over-crowding and non-car ownership. Townsend deprivation scores were derived by applying National Census data [26] based on validated residential postcode at the time of diagnosis to the patient cohort. Using this method, it was possible to ascertain area-level deprivation scores for 100% of subjects in the study (Table S3). We assigned deprivation score to the cases according to the lower super output area of residence at the time of diagnosis, using the index preceding year of diagnosis (1997–2000–1991 Census; 2001–2010–2001 Census; and 2011–2016–2011 Census). Townsend deprivation score was stratified into population-weighted fifths (I–V) [27] based on the total population of England at the time of each pertaining census [26] for calculation of 5-year survival rates by area-level deprivation.

### Statistical analysis

Varying trends in survival over time, by ethnic group and area-level deprivation (Townsend fifths I–V) were initially described using Kaplan–Meier estimation [28], for all cancers combined and each major diagnostic group (I, II, III, IV–XII).

Unadjusted, 5-year survival estimates were presented by 10-year time period of diagnosis (1997–2006, 2007–2016). These 10-year periods were preferable when exploring diagnostic-group-specific trends, due to small within-group case numbers. For analyses of all cancers combined, enhanced case numbers permitted the use of shorter 5-year time periods, which are presented in addition to provide a greater insight into survival trends over time.

Cox regression models [29] were used to determine prognostic disparities in outcome, after adjustment for other factors known to affect survival (Table S4); and, where present, whether inequities in survival changed over time for different cancer types (leukaemia, CNS tumours, and other solid tumours). In our paediatric cancer population, an even distribution of cases across the population-weighted fifths of deprivation would not be expected because there are more births and children living in more deprived areas compared to those in less deprived areas [30]. In all, 25.6% of the non-south Asian cases were assigned to the most deprived quintile V, whereas 68.2% of south Asian cases were in the most deprived areas. This mirrors the distribution of these ethnic groups in the general population [31]. When modelling these data, the effects of area-based socio-economic deprivation would therefore be confounded with ethnic group (Table 1a). In the presence of such structural confounding, it is not possible to distinguish between any associations due to the independent effects of area-based deprivation (at diagnosis) versus ethnicity [32]. Therefore, we modelled the overall joint effect of ethnicity and area-based deprivation at diagnosis on survival, framed through causal interpretation of the deprivation coefficient [32]. Goodness-of-fit testing using Bayesian Information Criterion [33] was performed for every single fully adjusted model to confirm that a linear term was more optimal than a non-linear functional form for modelling area-based Townsend deprivation.

**Table 1:** (a) The number of children by International Classification of Childhood Cancer (Third edition) diagnostic group, age, sex and Townsend fifths of deprivation (I–V) for south Asian and non-south Asian children diagnosed with cancer aged 0–14 years in Yorkshire (1997–2016), by 10-year period of diagnosis; (b) the number of children by International Classification of Childhood Cancer (Third edition) diagnostic group, age group and sex for 2674 children diagnosed with cancer aged 0–14 years in Yorkshire 1997–2016, by Townsend deprivation fifths^a^.

a						
Number of cases	Overall (1997–2016)		1997–2006		2007–2016	
*n* = 2674	Non-south Asian (*n* = 2441, 91.3%)	South Asian (*n* = 233, 8.7%)	Non-south Asian (*n* = 1174)	South Asian (*n* = 93)	Non-south Asian (*n* = 1267)	South Asian (*n* = 140)
*Diagnostic group*^b^						
Leukaemia (*n* = 805)	734 (91.2)	71 (8.8)	374 (92.4)	31 (7.6)	360 (90.0)	40 (10.0)
Lymphoid leukaemias (*n* = 673)	614 (91.2)	59 (8.8)	309 (92.2)	26 (7.8)	305 (90.2)	33 (9.8)
Acute myeloid leukaemia (*n* = 98)	93 (94.9)	5 (5.1)	49 (94.2)	3 (5.8)	44 (95.7)	2 (4.4)
Other (*n* = 34)	27 (79.4)	7 (20.6)	16 (88.9)	2 (11.1)	11 (68.8)	5 (31.3)
Lymphoma (*n* = 287)	253	34	142 (88.8)	18 (11.3)	111 (87.4)	16 (12.6)
Hodgkin lymphoma (*n* = 124)	108 (87.1)	16 (12.9)	64 (88.9)	8 (11.1)	44 (84.6)	8 (15.4)
Non-Hodgkin lymphoma (*n* = 149)	131 (87.9)	18 (12.1)	68 (87.2)	10 (12.8)	63 (88.7)	8 (11.3)
Miscellaneous lymphoma (*n* = 14)	14 (100)	0 (–)	10 (100.0)	0 (-)	4 (100.0)	0 (–)
CNS tumours (*n* = 668)	616 (92.2)	52 (7.8)	283 (95.0)	15 (5.0)	333 (90.0)	37 (10.0)
Ependymoma (*n* = 71)	65 (91.6)	6 (8.5)	24 (100.0)	0 (–)	41 (87.2)	6 (12.8)
Astrocytoma (*n* = 299)	276 (92.3)	23 (7.7)	124 (94.7)	7 (5.3)	152 (90.5)	16 (9.5)
Embryonal (*n* = 125)	116 (92.8)	9 (7.2)	66 (95.7)	3 (4.4)	50 (89.3)	6 (10.7)
Other glioma (*n* = 86)	81 (94.2)	5 (5.8)	36 (97.3)	1 (2.7)	45 (91.8)	4 (8.2)
Other (*n* = 87)	78 (89.7)	9 (10.3)	33 (89.2)	4 (10.8)	45 (90.0)	5 (10.0)
Other solid tumours^c^ (*n* = 914)	838	76	375 (92.8)	29 (7.2)	463 (90.8)	47 (9.2)
Nervous system (IV a, b) (*n* = 187)	172 (92.0)	15 (8.0)	72 (93.5)	5 (6.5)	100 (90.9)	10 (9.1)
Renal (VI a–c) (*n* = 149)	137 (92.0)	12 (8.0)	68 (93.2)	5 (6.7)	69 (90.8)	7 (9.2)
Bone (VIII a–e) (*n* = 115)	108 (93.9)	7 (6.0)	51 (92.7)	4 (7.3)	57 (95.0)	3 (5.0)
Soft tissue sarcoma (IX a–e) (*n* = 171)	164 (95.9)	7 (4.1)	76 (97.4)	2 (2.6)	88 (94.6)	5 (5.4)
Germ cell (X a–e) (*n* = 90)	84 (93.3)	6 (6.7)	45 (95.7)	2 (4.3)	39 (90.7)	4 (9.3)
Other (V, VIII a–c, XI a–f, XII a, b) (*n* = 202)^d^	173 (85.6)	29 (14.4)	63 (85.1)	11 (14.9)	110 (85.9)	18 (14.1)
*Deprivation at diagnosis*^a,e^						
Least deprived I (*n* = 416)	405 (16.6)	11 (4.7)	197 (16.8)	5 (5.4)	208 (16.4)	6 (4.3)
II (*n* = 456)	447 (18.3)	7 (3.0)	216 (18.4)	3 (3.2)	231 (18.2)	4 (2.9)
III (*n* = 485)	464 (19.0)	21 (9.0)	203 (17.3)	8 (8.6)	261 (20.6)	13 (9.3)
IV (*n* = 536)	501 (20.5)	35 (15.0)	252 (21.5)	12 (12.9)	249 (19.7)	23 (16.4)
Most deprived V (*n* = 783)	624 (25.6)	159 (68.2)	306 (26.1)	17 (69.9)	318 (25.1)	94 (67.1)
*Age at diagnosis (years)*^e^						
0–4 (*n* = 1277)	1155 (47.3)	122 (52.4)	534 (45.5)	45 (48.4)	621 (49.0)	77 (55.0)
5–9 (*n* = 698)	634 (26.0)	64 (27.5)	311 (26.5)	25 (26.9)	323 (25.5)	39 (27.9)
10–14 (*n* = 699)	652 (26.7)	47 (20.2)	329 (28.0)	23 (24.7)	323 (25.5)	24 (17.1)
*Sex*^e^						
Male (*n* = 1483)	1355 (55.5)	128 (54.9)	676 (57.6)	52 (55.9)	679 (53.6)	76 (54.3)

b					
Number of cases	Townsend deprivation fifths^a^				
*n* = 2674	I	II	III	IV	V
*Diagnostic group*^b^					
Leukaemia (*n* = 805)	122 (15.2)	139 (17.3)	153 (19.0)	165 (20.5)	226 (28.1)
Lymphoid leukaemias (*n* = 673)	105 (15.6)	122 (18.1)	131 (19.5)	127 (18.9)	188 (27.9)
Acute myeloid leukaemia (*n* = 98)	10 (10.2)	14 (14.3)	19 (19.4)	28 (28.6)	27 (27.6)
Other (*n* = 34)	7 (20.6)	3 (8.8)	3 (8.8)	10 (29.4)	11 (32.4)
Lymphoma (*n* = 287)	40 (13.9)	48 (16.7)	50 (17.4)	56 (19.5)	93 (32.4)
Hodgkin lymphoma (*n* = 124)	18 (14.5)	18 (14.5)	15 (12.1)	31 (25.0)	42 (33.9)
Non-Hodgkin lymphoma (*n* = 149)	22 (14.8)	27 (18.1)	30 (20.1)	22 (14.8)	48 (32.2)
Miscellaneous lymphoma (*n* = 14)	–	3 (21.4)	5 (35.7)	3 (21.4)	3 (21.4)
CNS tumours (*n* = 668)	108 (16.2)	117 (17.5)	122 (18.3)	129 (19.3)	192 (28.7)
Ependymoma (*n* = 71)	11 (15.5)	14 (19.7)	15 (21.1)	9 (12.7)	22 (31.0)
Astrocytoma (*n* = 299)	40 (13.4)	52 (17.4)	55 (18.4)	58 (19.4)	94 (31.4)
Embryonal (*n* = 125)	26 (20.8)	21 (16.8)	21 (16.8)	27 (21.6)	30 (24.0)
Other glioma (*n* = 86)	14 (16.3)	16 (18.6)	19 (22.1)	15 (17.4)	22 (25.6)
Other (*n* = 87)	17 (19.5)	14 (16.1)	12 (13.8)	20 (23.0)	24 (27.6)
Other solid tumours^c^ (*n* = 914)	146 (16.0)	150 (16.4)	160 (17.5)	186 (20.4)	272 (29.8)
Nervous system (IV a, b) (*n* = 187)	35 (18.7)	27 (14.4)	36 (19.3)	34 (18.2)	55 (29.4)
Renal (VI a–c) (*n* = 149)	26 (17.5)	29 (19.5)	19 (12.8)	29 (19.5)	46 (30.9)
Bone (VIII a–e) (*n* = 115)	22 (19.1)	16 (13.9)	22 (19.1)	24 (20.9)	31 (27.0)
Soft tissue sarcoma (IX a–e) (*n* = 171)	30 (17.5)	37 (21.6)	27 (15.8)	38 (22.2)	39 (22.8)
Germ cell (X a–e) (*n* = 90)	9 (10.0)	12 (13.3)	24 (26.7)	19 (21.1)	26 (28.9)
Other (V, VIII a–c, XI a–f, XII a, b) (*n* = 202)^d^	24 (11.9)	29 (14.4)	32 (15.8)	42 (20.8)	75 (37.1)
*Age at diagnosis (years)*					
0–4 (*n* = 1277)	170 (13.3)	213 (16.7)	231 (18.1)	256 (20.1)	407 (31.9)
5–9 (*n* = 698)	123 (17.6)	116 (16.6)	131 (18.8)	142 (20.3)	186 (26.7)
10–14 (*n* = 699)	123 (17.6)	125 (17.9)	123 (17.6)	138 (19.7)	190 (27.2)
*Sex*					
Male (*n* = 1483)	199 (16.7)	189 (15.9)	211 (17.7)	249 (20.9)	343 (28.8)

*CNS* central nervous system.

^a^Townsend deprivation scores [25] used as a measure of area-level deprivation; assigned to population-weighted fifths (of England’s total population) for analysis where I = least deprived and V = most deprived.

^b^Diagnoses were categorised according to the International Childhood Cancer Classification system, third edition (ICCC-3) [23].

^c^Other solid tumours group includes diagnostic categories corresponding to ICCC codes IV–XII.

^d^Top five most common ‘Other’ cancers (V, VIII a–c, XI a–f, XII a, b): retinoblastoma (49.0%), hepatoblastoma (13.9%), other and unspecified carcinomas (11.4%), thyroid carcinomas (10.9%) and hepatic carcinomas (5.0%).

^e^The percentages for deprivation, age and sex are % by those variables within ethnic group (south Asian/non-south Asian).

Cox models were re-parameterised to provide the estimated effect of area-level deprivation on survival (continuous Townsend index score), for each 5-year time period of diagnosis [34]. Hazard ratios (HRs) were reported from the Cox regression models and represent the increase in the expected log of the relative hazard, or risk of mortality for each one unit increase in Townsend score, where increasing Townsend score is indicative of greater area-level deprivation [25]. Outcomes for children with lymphomas were modelled according to 10-year time periods due to the small number of deaths within this diagnostic group.

All HRs for each covariate were mutually adjusted for confounding based on the minimal sufficient adjustment set for estimating the total effect of area-based deprivation at diagnosis on survival outcome. The minimal sufficient adjustment set for modelling the effect of area-based deprivation on childhood cancer survival at each time point was informed by causal inference methods [35] (Fig. S1) using directed-acyclic graphs (DAG) within DAGitty software Version 3.0 [36]. Ethnic group (south Asian, White, other) was adjusted for in each model, together with the other factors relating to patient case-mix and clinical management (sex, age at diagnosis, relapse, ICCC-3 subgroup, stage of disease and treatment at a PTC). Sensitivity analyses was performed with each model being run on cases diagnosed in non-south Asian children only.

In instances where data on stage at diagnosis were unavailable (leukaemia 5.7%; lymphoma 42.2% (Hodgkin lymphoma: 18.6% missing; non-Hodgkin lymphoma 58.4% missing), CNS 3.3%, other solid tumours; 75.0%), these were assumed to be missing at random and imputed using ordered logistic regression. Table S1 presents the proportion of missing stage and grade information by ethnic group (south Asian/non-south Asian) and area-based deprivation for each major ICCC-3 diagnostic group. We generated a total of 50 imputed data sets for each diagnostic group (I, II, III, IV–XII) and all cancers combined. Each imputation model included all previously listed variables, in addition to binary indicators for treatment, surgery, radiotherapy and chemotherapy. To avoid underestimation of stage and survival time, the Nelson–Aalen estimate for the cumulative hazard function [37] and a death indicator [38] were included in each imputation model. All imputation methods were implemented in Stata 16 [39], using multiple imputation by chained equations [40].

Schoenfeld residuals were used to assess the Cox proportional hazard assumption for each imputation [41] whereby random scatters around zero on plots of the residuals against the rank survival time for each covariate validated these assumptions [42]. The models for all cancers combined, lymphomas and other solid tumours violated the proportional hazards assumption for age at diagnosis; for these models, age was included as a time-dependent variable. Relapse was included as a time-dependent variable in all models. Monte Carlo standard errors were calculated to quantify the level of uncertainty in all estimated quantities of each model, and the c-index measure of discrimination was used to assess predictive performance [43]. Results from complete case analyses were compared to the results from the multiple imputation models to check there were no major differences in terms of the directionality of effects. Sensitivity analyses was also performed to compare results from multiply imputed models with models including all cases but with an extra category for ‘unknown’ added for all variable where data were incomplete for some cases. A reduction in Monte Carlo standard errors were indicative of an overall improvement in the precision of the analysis when using multiple imputation by chained equations models. Only results for multiply imputed models are presented.

## Results

Table 1a, b present the sociodemographic profiles of the 2674 children included in the analyses. Table S2 presents the HES ethnic groups of all children in the cohort, according to 1991 and 2001 Census categories. The cohort was comprised of 85.8% White, 8.7% south Asian and 5.6% children of ‘other’ ethnic backgrounds, including *n* = 28 (1.1%) Black, *n* = 32 (1.2%) other Asian and *n* = 20 (0.7%) mixed background. Crude 5-year survival estimates are shown in Table 2a, b, by ethnic group and Townsend deprivation fifths (I–V), respectively.

**Table 2:** (a) Overall 5-year crude survival estimates (%) by ethnic group and period of diagnosis; for all cancers combined and major International Classification of Childhood Cancer (Third edition) diagnostic groups for children diagnosed with cancer aged 0–14 years in Yorkshire between 1997 and 2016^a^. (b) overall 5-year crude survival estimates (%) by area-based deprivation and period of diagnosis; for all cancers combined and major ICCC-3 groups (leukaemia, lymphoma, central nervous system tumours and other solid tumours) for children diagnosed with cancer aged 0–14 years in Yorkshire between 1997 and 2016^a^.

a					
		Period of diagnosis			
		1997–2006		2007–2016	
ICCC-3 diagnostic group^b^	Ethnicity	1997–2001	2002–06	2007–2011	2012–2016
All cancers combined (5-year periods)	Non-south Asian	74.0 (70.2–77.4)	79.7 (76.3–82.7)	80.9 (77.5–83.8)	85.2 (82.2–87.8)
	South Asian	64.4 (48.7–76.5)	81.3 (67.1–89.8)	75.4 (62.1–84.7)	80.7 (70.1–87.9)
All cancers combined	Non-south Asian	77.0 (74.4–79.3)		83.1 (80.9–85.1)	
	South Asian	73.1 (62.9–80.9)		78.5 (70.6–84.5)	
Leukaemia	Non-south Asian	82.3 (78.0–85.8)		86.4 (82.4–89.6)	
	South Asian	74.2 (55.0–86.2)		87.0 (71.6–94.3)	
Lymphoma	Non-south Asian	87.2 (80.5–91.8)		96.3 (90.3–98.6)	
	South Asian	72.2 (45.6–87.4)		81.3 (52.5–93.5)	
CNS tumours	Non-south Asian	71.6 (66.0–76.5)		78.8 (73.9–82.3)	
	South Asian	60.0 (31.8–79.7)		63.9 (46.1–77.2)	
Other solid tumours^c^	Non-south Asian	71.7 (66.8–76.0)		80.6 (76.6–83.9)	
	South Asian	79.3 (59.6–90.1)		81.8 (66.9–90.5)	

b					
ICCC-3 diagnostic group^b^	Deprivation^d^	Period of diagnosis			
		1997–2006		2007–2016	
		1997–2001	2002–2006	2007–2011	2012–2016
All cancers combined	I	73.3 (63.5–80.8)	81.2 (72.1–87.6)	81.1 (71.4–87.8)	81.3 (72.9–87.3)
	II	85.3 (76.8–90.9)	80.2 (71.7–86.4)	83.0 (74.4–88.9)	88.9 (82.0–93.3)
	III	76.0 (66.3–83.2)	81.1 (72.5–87.2)	82.1 (74.7–87.6)	84.5 (77.1–89.7)
	IV	71.1 (62.1–78.1)	75.2 (67.2–81.5)	77.2 (68.4–83.9)	84.8 (78.1–90.0)
	V	66.8 (59.6–73.1)	81.7 (75.2–86.6)	79.4 (73.3–84.3)	85.0 (79.0–89.4)
All cancers combined	I	77.2 (70.8–82.4)		81.2 (75.2–85.9)	
	II	82.6 (76.9–87.0)		86.2 (81.0–90.0)	
	III	78.7 (72.5–83.6)		83.3 (78.3–87.3)	
	IV	73.3 (67.5–78.2)		81.6 (76.4–85.8)	
	V	74.1 (69.3–78.3)		82.0 (77.9–85.5)	
Leukaemia	I	89.5 (79.3–94.9)		86.7 (74.1–93.4)	
	II	87.8 (77.9–93.5)		82.5 (70.7–89.9)	
	III	82.8 (71.1–90.1)		85.2 (75.9–91.1)	
	IV	76.2 (65.6–83.9)		90.0 (81.0–94.9)	
	V	76.5 (67.7–83.3)		87.0 (79.1–92.1)	
Lymphoma	I	86.4 (63.4–95.4)		88.5 (61.4–97.0)	
	II	–		–	
	III	76.9 (55.7–88.9)		–	
	IV	89.3 (70.4–96.4)		89.3 (70.4–96.4)	
	V	80.4 (67.3–88.6)		93.9 (77.9–98.5)	
CNS tumours	I	70.8 (55.8–81.6)		72.9 (59.6–82.4)	
	II	80.9 (66.4–89.5)		87.0 (76.4–93.0)	
	III	75.5 (61.5–85.0)		78.8 (66.8–86.9)	
	IV	60.0 (47.1–70.7)		74.0 (61.0–83.2)	
	V	71.4 (60.5–79.8)		75.2 (65.8–82.4)	
Other solid tumours^c^	I	66.2 (53.3–76.2)		82.0 (71.5–88.9)	
	II	71.4 (59.3–80.5)		84.8 (74.8–91.1)	
	III	77.9 (66.1–86.1)		80.4 (70.7–87.1)	
	IV	75.3 (64.7–83.1)		77.3 (67.6–84.4)	
	V	70.5 (61.1–78.1)		80.6 (73.3–86.1)	

*ALL* acute lymphoblastic leukaemia, *CNS* central nervous system, – no deaths recorded.

^a^For those with multiple tumours (*n* = 40), only first recorded tumour was included in the calculation of 5-year survival rates.

^b^Diagnoses were categorised according to the International Childhood Cancer Classification system, third edition (ICCC-3) [23].

^c^Other solid tumours group includes diagnostic categories corresponding to ICCC codes IV–XII.

^d^Townsend deprivation scores [25] used as a measure of area-level deprivation; assigned to population-weighted fifths (of England’s total population) for analysis where I = least deprived and V = most deprived.

South Asian individuals were allocated a considerably higher median Townsend score (4.9 (interquartile range (IQR): 2.1–6.8) compared to non-south Asians (−0.2 (IQR: −2.5–3.3)). Similarly, 68.2% of south Asian children fell into the most deprived fifth. Non-south Asians accounted for 25.6% of this group (V).

**Fig. 1:**
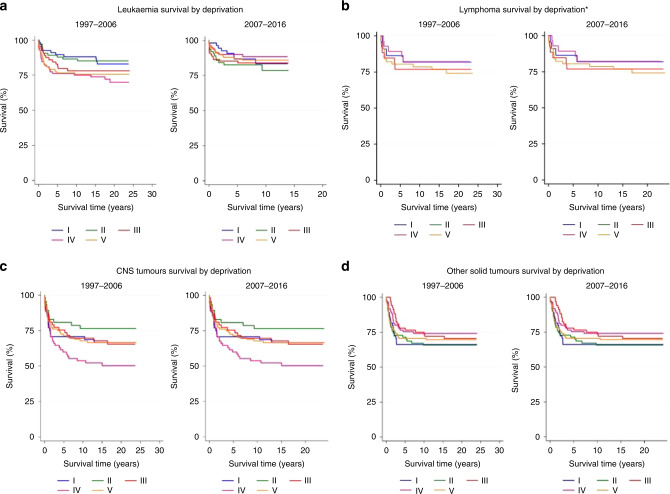
Kaplan–Meier survival estimates of children (0–14 years) diagnosed with cancer whilst resident in the Yorkshire & Humber region between 1997 and 2016. Survival estimates presented separately for the following diagnostic groups **a** leukaemia, **b** lymphoma, **c** CNS tumours and **d** other solid tumours, stratified by Townsend deprivation fifths (I–V) and time period of diagnosis: 1997–2006 and 2007–2016.

Long-term survival estimates according to Townsend fifths of deprivation are given in Table 2b by period of diagnosis and ICCC-3 diagnostic group. We observed a clear social gradient in the 5-year survival estimates of children diagnosed with leukaemia (including ALL) between 1997 and 2006 (Tables 2b and S5a, b and Fig. 1). The probability of surviving at least 5 years following a leukaemia diagnosis was almost 13% greater for children in the least deprived fifth (I) (89.5%) compared to children in the two most deprived fifths (IV: 76.2% and V: 76.5%) (Table 2b).

**Fig. 2:**
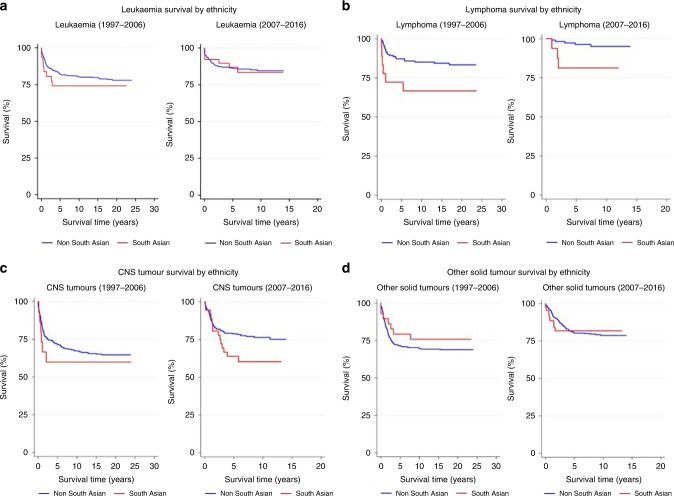
Kaplan–Meier survival estimates of children (0–14 years) diagnosed with cancer whilst resident in Yorkshire & the Humber region between 1997 and 2016. Survival estimates presented separately for the following diagnostic groups **a** leukaemia, **b** lymphoma **c** CNS tumours and **d** other solid tumours, by ethnic group (non-south Asian and south Asian) and time period of diagnosis: 1997–2006 and 2007–2016.

Concordant disparities were apparent when the cohort was stratified by ethnic group (Table 2a and Fig. 2); the 5-year survival rate for south Asian children diagnosed with leukaemia between 1997 and 2006 (72.2%) was 15% lower than the estimate for non-south Asians diagnosed during that time (87.2%). This pattern altered over time (Fig. 1a), with survival estimates rapidly improving by almost 13.0% for south Asian children (Table 2a), compared with an increase of just 4.1% for non-south Asian children (2007–2016; non-south Asian 5-year survival: 86.4% versus 87.0% south Asian). We observed a similar pattern of converging survival estimates across Townsend fifths; with large improvements in the long-term survival of children in fifths IV and V, coupled with negligible improvements for children in fifths I, II and III during this time period. These changes led to a survival advantage for children in the most deprived fifths diagnosed with leukaemia up until 2016 (IV: 90.0% V: 87.0%) compared to their more affluent peers (I: 86.7% II: 82.5%).

Evidence of widening differences in long-term survival rates were observed for children diagnosed with lymphoma across the study period (Fig. 2b). Non-south Asian children were 10% more likely to survive for at least 5 years after their diagnosis (87.2%), compared to south Asian children (72.2%) (1997–2006). Survival improved for both ethnic groups over the 10-year period (Fig. 2b); however, a 15% gap in survival was present for diagnoses of lymphoma between 2007 and 2016 (Table 2a). These ethnic disparities in lymphoma survival were even more pronounced when children with non-Hodgkin lymphoma (NHL) were analysed separately (Table S5a). Five-year survival estimates were almost 35% higher for non-south Asians diagnosed with NHL between 2007 and 2016, compared to south Asians. Clear trends were less evident when the lymphoma cohort was stratified by Townsend fifths (Table 2b).

The 5-year survival estimate for south Asian children diagnosed with a CNS tumour between 1997 and 2006 (60.0%) was almost 12% lower than the equivalent estimate for non-south Asians diagnosed during that time (71.6%). This difference was more pronounced (15% lower) for south Asian (63.9%) and non-south Asian children (78.8%) diagnosed during the most recent period, 2007–2016. Despite consistent increases in survival estimates across each deprivation fifth from 1997–2006 to 2007–2016, we found considerable evidence of socio-economic differences in CNS tumour survival which persisted across both time periods. There was a stark difference in survival estimates between children in the least (I) and second-least deprived (II) fifth: with children in the least deprived fifth (I) around 10% (1997–2006) and 15% (2007–2016) more likely to die within 5 years of their diagnosis compared to children in group II. Children from areas of low/intermediate levels of deprivation (II and III) had a greater probability of surviving at least 5 years after their diagnosis when compared to children in the most deprived fifths (IV and V) (Table 2b).

Other non-CNS solid tumours were the only diagnostic group where south Asian children had higher 5-year survival (79.3%) in the first diagnostic period (1997–2006), compared to non-south Asian children (71.7%). These ethnic differences reduced considerably over time, largely driven by a 9% increase in 5-year survival for non-south Asian children. Only a 1.2% difference in survival remained between south Asian (81.8%) and non-south Asian children (80.6%) diagnosed with other solid tumours between 2007 and 2016. There was no evidence of a social gradient in 4-year survival for other solid tumours diagnosed between 1997 and 2006. Five-year survival was highest in children in fifths V (77.9%) and IV (75.3%). Subtle evidence of a social gradient was apparent for children diagnosed in the latest 10-year period (2007–2016) whereby children in the least deprived fifths (I: 82.0% II: 84.8%) had more favourable long-term survival estimates compared to those in the most deprived fifths (IV: 77.3%: V: 80.6%).

### Cox multivariable regression

The results of multivariable Cox proportional hazards regression modelling for all cancers combined and each main diagnostic group are presented in Table 3.

**Table 3:** Hazard ratios (HRs) and 95% confidence intervals (CIs) from Cox regression models presenting the association between increasing area-based deprivation^a^ and risk of death in children (aged 0–14 years) with a diagnosis of all cancers, leukaemia, central nervous system tumours or other solid tumours resident in Yorkshire & the Humber between 1997 and 2016.

	Year of diagnosis							
	1997–2001		2002–2006		2007–2011		2012–2016	
ICCC-3 diagnostic group	HR^b^	95% CI	HR^b^	95% CI	HR^b^	95% CI	HR^b^	95% CI
All cancers combined	1.05	1.00–1.09	1.00	0.95–1.06	1.02	0.97–1.07	1.00	0.96–1.07
I. Leukaemia^c^	1.11	1.03–1.20	0.95	0.87–1.04	0.94	0.84–1.04	1.04	0.94–1.14
II. Lymphoma^c^	1.01	(0.93–1.20)			1.04	(0.85–1.28)		
III. CNS tumours	1.02	0.95–1.01	1.07	0.98–1.16	1.08	1.00–1.16	0.96	0.88–1.15
IV–XII. Other solid tumours	1.04	0.97–1.12	1.00	0.91–1.10	1.02	0.94–1.10	1.07	0.98–1.16

All models derived from multiple imputation to account for missing staging diagnostic data.

^a^Townsend deprivation scores [25] used as a measure of area-level deprivation based on validated postcode at diagnosis. Hazard ratios present the increase in expected mortality risk with one unit increase in Townsend score, where increasing Townsend score is indicative of greater material deprivation. Hazard ratios are presented at each time period of diagnosis—to allow us to look at changes in trends over time.

^b^All HRs are mutually adjusted for confounding based on the minimal sufficient adjustment set for estimating the direct area-based deprivation on survival outcome over time, accounting for prognostic factors and patient case-mix (see Table S4 for full model details).

^c^Model for leukaemia and lymphoma do not include level of treatment at principal treatment centre as care was standardised across study period. 97.5% received all treatment for leukaemia at PTC and 94.7% for lymphomas.

For all cancers combined, children living in more deprived areas at the time of diagnosis were significantly more likely to die (HR: 1.05 (1.00–1.09) when diagnosed between 1997 and 2001. However, we found no evidence of notable socio-economic differences in mortality risk for paediatric cancers diagnosed from 2002 onwards in Yorkshire (Table 3).

In line with univariable estimates (Table 2b), a more deprived background was positively associated with an excess risk of death for children diagnosed with leukaemia (HR: 1.11 (1.03–1.20)) before 2002. Socio-economic differences in prognosis observed during this time were further exaggerated when ALL cases were considered alone; whereby a one unit increase in Townsend score was associated with a 17% increase in expected hazard (HR: 1.17 (1.07–1.28). Prognostic outcomes improved substantially for children living in more deprived areas at the time of their diagnosis between 2002 and 2011, whereby increasing levels of material deprivation were associated with a reduced risk of death (HR range 0.94–0.95, Table 3). There was considerable variation in survival for children diagnosed with all leukaemias combined (HR: 1.04 (0.94–1.14)) between 2012 and 2016, by area-based deprivation. Equivalent trends were not observed when ALL was modelled separately (HR: 0.97 (0.86–1.09) (Table S6).

We observed no notable socio-economic differences in survival for lymphomas diagnosed across the study period. However, we found evidence of widening socio-economic disparities for children diagnosed with CNS tumours between 1997 and 2011. Excess risk of death conferred by living in more materially deprived areas at the time of diagnosis was greatest for children diagnosed between 2007 and 2011 (HR: 1.08 (1.0–1.16)). Estimates for children diagnosed in the latest diagnostic time period (HR: 0.96 (0.88–1.15)) were indicative of reducing variation in CNS tumour survival for cases diagnosed from 2012 onwards. An equivalent trend was observed when high grade CNS tumours were modelled separately (Table S6). Socio-economic differences in mortality were apparent for those diagnosed with low grade CNS tumours before 2001 (1.05 (0.93–1.20) and between 2007 and 2011 (1.09 (0.94–1.26).

Socio-economic differences were apparent for children diagnosed with other non-CNS solid tumours between 1997 and 2001 (HR: 1.04 (0.97–1.12)). Survival advantages for children resident in less deprived areas at the time of their diagnosis persisted across the study period (2012–2016 HR: 1.07 (0.98–1.16)).

## Discussion

We describe a novel population-based study estimating trends in socio-economic and ethnic variation in childhood cancer survival in a region of the UK over a 20-year period. A long-term trend of improvement in survival rates from childhood cancer was observed. The most substantial improvements in overall survival were observed for south Asian children, and those living in the most deprived areas. Some of these improvements in outcome will have led to reduced ethnic and socio-economic differences in survival across these groups. However, there remained scope for improvement as variation remained for certain cancer types. Consistent and persisting disparities in overall survival from paediatric lymphoma were identified from univariable estimates whereby south Asian children had a 15% increased risk of death within the 5 years after their diagnosis compared to their non-south Asian peers. This trend was observed across the study period. We found an excess risk in mortality for children from the more deprived backgrounds diagnosed with CNS tumours in Yorkshire up until 2012, and other solid tumours as late as 2016 that persisted after adjustment for confounding.

Utilising data from a specialist, regional population-based register provided comprehensive information from across the child life course covering initial diagnostic and treatment information and detailed follow-up information, to a level of detail and quality which is not widely held in national cancer registries over the time period of this study [44]. Exploitation of this rich data source meant it was possible to account for clinical and prognostic features and confounders in the regression modelling analysis known to be important determinants of survival, such as tumour stage and grade, detailed sub-type and disease recurrence [15].

The socio-economic distribution of the childhood cancer population in Yorkshire, such that south Asian children were more likely to live in more deprived areas, meant that it was not possible to model the direct effect of ethnicity over time in our cohort [30]. This inherent structural confounding within the population makes it impossible to isolate the true causal ‘effect’ of ethnicity. Whether we were to consider physical phenotype, patient perception by others, genetic background or cultural beliefs, singly or jointly, all of these would likely be correlated with area-based deprivation at the time of diagnosis. Nonetheless, overall survival rates have been presented by ethnic group with the aim of elucidating the potential role of ethnicity in explaining in part the drivers of observed patterns in survival outcome. These estimates were further supplemented by results from multivariable models, fully adjusting for confounding [30, 33].

Around two-thirds of the study cohort were treated in the Leeds PTC, with the rest receiving their treatment at the Sheffield Children’s Hospital. South Asian children accounted for 11% of patients treated in Leeds. Whereas south Asian children comprised 4% of children receiving treatment in the Sheffield PTC. Importantly, there were no ethnic differences in the likelihood of receiving specialist care; 89% of non-south Asians in the study cohort received care at a PTC compared to 90% of south Asians. The care provided at the two PTCs in Yorkshire is likely to be homogeneous for the paediatric cancer population (0–14 years). Both PTCs adhere to common treatment guidelines, tumour specific signposting and enrolment of patients in national and international clinical trials as appropriate, in order to ensure the highest standards of care.

Our findings of higher overall survival among the least deprived childhood cancer population and non-south Asian children diagnosed with ALL between 1997 and 2006 in Yorkshire are consistent with findings from previous population-based UK studies [12–14]. However, the present study has demonstrated that the pattern of disparities in childhood leukaemia survival has changed substantially over time. We observed a novel trend towards reducing disparities between 2002 and 2016 showing high survival estimates for both south Asian children and those living in more deprived areas when diagnosed from 2002 onwards. Similarly, a recent multi-national genomics study [45] reported the highest 5-year overall survival rates for south Asian children with ALL (98.2%), compared to White (95.5%), Black (89%) and other ethnic groups [46]. The authors emphasise the important role of genetic ancestry in ALL treatment outcomes and found striking differences of ALL subtypes across ancestries. South-east Asian genetic ancestry was positively associated with DUX4-rearrangements, a sub-group commonly associated with favourable prognosis [47]. This points to a potential genetic basis for some of the racial disparities in ALL survival that may, in part, provide an explanation for the observed findings in the present study but requires further investigation.

A large-scale UK study of children diagnosed with cancer between 1981 and 1996 found little evidence of ethnic differences in survival outcome for most of the diagnostic groups studied [14]. In line with findings from the current study, evidence of a poorer prognosis for south Asian children diagnosed with ALL before 1996 was found. The authors attributed these ethnic disparities in ALL survival to potential ethnic variations in drug metabolism, susceptibility to infection or compliance with therapy. Previous ALL trials have noted an increased rate of death in remission for Asian children [48], which could also contribute to observed differences in survival in this and the current study. Future studies should use linked Office for National Statistics (ONS) mortality data to facilitate an in-depth exploration into the cause and timing of death in relation to treatment across different ethnic groups.

The reducing but nevertheless persisting disparities in survival outcome by deprivation for childhood CNS tumours are a novel finding in the UK. Children living in the most deprived areas at the time of a CNS tumour diagnosis up until 2012 were considerably more likely to die than comparable children living in areas of lower material deprivation. This also warrants further examination. Understanding the underlying mechanisms is not trivial as they are likely to differ between childhood cancer types and healthcare settings. While socio-economic disparities in survival for children with CNS tumours have been observed in Swiss [49], Swedish [50], and Finnish studies [51], much of the existing UK evidence base has focused on leukaemia [12–14] and evidence for other paediatric cancers is lacking. Potential explanations for the observed social and ethnic variations in childhood cancer survival mainly relate to differences in treatment protocols and adherence [13]. There is a possibility that prognostic disparities observed in some studies are associated with ethnic/social differences in the access and time to treatment, leading to more advanced-stage disease presentation [49], or varying incidence of sub-types of cancers with differing prognosis. Unlike many previously published studies, we were able to take detailed sub-type and staging into account. We conducted a sensitivity analysis modelling high and low-grade CNS tumours separately. This resulted in similar conclusions for CNS tumours overall in both high and low grade tumours. Adjustment for histopathological group did not change our results.

Survival differences persisted for south Asians with lymphoma in contrast to non-south Asians. The observed disparities in lymphoma survival are in line with findings from a previously published study in Yorkshire [15] and appear to be driven by the disproportionately poor prognosis of NHL in south Asian children (Fig. S2) (Table S5a). Once data from universal fine scale mapping and genomic sequencing of individuals becomes available [52]; future work should explore the extent to which these observed trends can be linked to differences in allelic variations and cancer susceptibility (constitutional mismatch repair deficiency and other cancer predisposition syndromes) amongst disparate ethnic groups [53].

It has been argued that ethnic differences in cancer survival could simply be socio-economic differences under a different guise [54]. To our knowledge, there have been no comparable studies conducted within the Indian sub-continent which report on within-group survival rates across the south Asian population. Supplementing the Townsend index with other individual measures of socio-economic status (SES) which incorporate income, education and occupation [12] may provide further insight into the intricacies of SES, ethnicity and childhood cancer survival beyond those of neighbourhood and ecological contextual effects. Nevertheless, our findings provide important benchmarking information for the Public Health England Public Health Outcomes Framework [8], which aims to pool recent data to support the understanding of inequalities in health for different populations in England. At present, the Public Health Outcomes Framework report [8] does not examine inequalities in cancer mortality by ethnic group, as this is not recorded on UK death registration records [8].

National cancer registries routinely produce reports which include overall survival estimates for England, as well as trends in survival [1] but do not account for demographic factors known to impact upon inequalities. Findings from this study demonstrate the vital importance of considering the joint impact of ethnicity and socio-economic deprivation on childhood cancer survival at a national level. Only national cancer registries have the breadth of information required to facilitate more detailed sub-analyses, which is key to identifying the underlying reasons for the disparities in survival observed in this study.

We acknowledge that in opting for an upper age limit of their 15th birthday, we have failed to consider outcomes for older children aged 15–16 years at diagnosis who will have received care in a paediatric setting. However, we felt that this facilitated a better comparison of our data with previously published UK outcomes data covering the 0–14 year age range. Patterns in survival for the older teenage and young adult age range will be explored in future work.

Further limitations of the work include the relatively small number of south Asians in our study and therefore a consequential loss of statistical power. To retain statistical power we considered broad, binary ethnic groups and area-level deprivation indicators but were unable to model outcomes of 2001 Census ethnic groups such as Pakistani, or Black populations separately due to small registration numbers. The relatively small numbers in this sample limited the extent to which we could perform further sub-group and sensitivity analysis, looking at survival trends by CNS sub-groups or exploring a differential effect of deprivation across ethnic groups (for example, the more deprived south Asians diagnosed in 1997–01 compared to the more deprived non-south Asians diagnosed in same time period). The most recent time periods have a lower statistical power due to increased survival rates [1] which could have contributed to the significant variances seen in earlier periods, but not for the most recent time periods. However, we did attempt to mitigate this by considering 10-year time periods when modelling outcomes for lymphomas, where the number of deaths were small. Given the differences in changes over time between different types of tumours, the models for all cancers combined should be interpreted with caution. Finally, staging information was unavailable for 32% of cases overall and 75% of other solid tumours (Table S1). The assumption that stage was missing at random is also likely not valid given that recording can vary by treatment site and tumour type, thus invoking potential bias. However, there were no major differences in the directionality of effects when complete case analyses were compared to the results from the multiple imputation models. Proportion of missing stage information was also similar across ethnic groups and Townsend quintiles.

Despite these limitations, the strengths of this analysis include use of fully adjusted multivariable effect estimates informed by causal inference methods to account for confounding and high data quality on ethnicity and deprivation. While HES ethnicity was missing for *n* = 174 individuals (and Onomap naming algorithms used), excluding these individuals from analyses did not have any major impact on model outputs (Table S7). We avoided using certain deprivation indices which are partially derived from a health component (e.g. Index of Multiple Deprivation) and are therefore likely to show artificial associations with any health outcomes such as survival. Moreover, the Townsend Index is a comparable measure of deprivation over time whereas the various Index of Multiple Deprivations are only applicable at single points in time [27].

In this study, we use the term inequalities to indicate any differences in survival outcome. Limited available evidence suggests that observed inequalities may arise during the childhood cancer pathway due to genetic differences, for example ethnic variations in drug metabolism, susceptibility to infection, compliance with therapy [48], or non-biological factors such as varying access to healthcare or potential discriminatory bases. If any disparities were present, this would merely indicate that ethnicity/socio-economic status and paediatric survival outcome are correlated in the population under study, at a given point in time. Findings from this descriptive study should prompt further investigation using randomised trials, which can assess specific aspects of an ‘effect’ of ethnicity and further elucidate potential structural or fundamental drivers of these observed differences.

This is the first study to evaluate health equity of children diagnosed with *all* paediatric malignancies in the past 15–20 years [16]. We provide a complete and current picture of trends in socio-economic and ethnic variation in childhood cancer survival in Yorkshire and conclude that even in the UK, with a universally accessible healthcare system, socio-economic and ethnic inequalities in childhood cancer survival exist. We reveal differential underlying patterns of inequalities and potential variation in the underlying mechanisms between types of childhood cancer. These data highlight the urgent need for a change in practice so that national cancer registration and analytic services report outcomes in relation to ethnicity and deprivation data in all analyses and reports.

## Supplementary information

Socio-economic and ethnic disparities in childhood cancer survival, Yorkshire, UK

## Data Availability

The data are not publicly available due to privacy or ethical restrictions. The data that support the findings of this study are available on request from the corresponding author (subject to review, with the appropriate ethical and information governance approvals).
