# Supplementary material for: Socio-economic and ethnic disparities in childhood cancer survival, Yorkshire, UK
Source: Br J Cancer. 2023 Feb 24;128(9):1710–22. doi: 10.1038/s41416-023-02209-x (PMC10133387; doi:10.1038/s41416-023-02209-x)
Supplement: Supplementary file 1 — Socio-economic and ethnic disparities in childhood cancer survival, Yorkshire, UK [file 41416_2023_2209_MOESM1_ESM.docx]

**Socioeconomic and ethnic disparties in childhood cancer survival, Yorkshire, UK**

**Supplementary material**

**Table S1.** Missing stage and grade information by ethnic group (south Asian/non-south Asian) and area-based deprivation for each major International Classification of Childhood Cancer (third edition) (ICCC-3) diagnostic group.

| % Missing stage/grade information | ICCC-3 Diagnostic group | | | |
| --- | --- | --- | --- | --- |
|  | **Leukaemia** | **Lymphoma** | **CNS tumours** | **Other solid tumours** |
| Overall | **46 (5.7)** | **121 (42.2)** | **22 (3.3)** | **676 (75.0)** |
| Ethnic group |  |  |  |  |
| Non-south Asian | 42 (5.7) | 108 (42.7) | 22 (3.6) | 616 (73.5) |
| South Asian | 4 (5.6) | 13 (38.2) | 0 (-) | 60 (79.0) |
| Deprivation |  |  |  |  |
| I | 9 (7.4) | 13 (32.5) | 1 (0.9) | 101 (69.2) |
| II | 9 (6.5) | 21 (43.8) | 5 (4.3) | 105 (70.0) |
| III | 8 (5.2) | 28 (56.0) | 6 (4.9) | 120 (75.0) |
| VI | 11 (6.7) | 21 (37.5) | 6 (4.7) | 145 (78.0) |
| V | 9 (4.0) | 38 (40.9) | 4 (2.1) | 205 (75.4) |

**Table S2.** Hospital episode statistics (HES) ethnic groups^1^ of n=2,674 children (0-14 years) diagnosed with cancer in Yorkshire between 1997 and 2016, according to 1991 and 2001 Census categories.

| Ethnic group (n=2,674) | 1991 Census (n, %) | 2001 Census (n, %) |
| --- | --- | --- |
| South Asian (n=233, 8.7) | 4 = Indian (n=23, 0.9) | H = Indian (Asian or Asian British) (n=23, 10.3) |
|  | **5 = Pakistani (n=140, 5.2)** | J = Pakistani (Asian or Asian British) (n=140, 59.8) |
|  | **6 = Bangladeshi (n=9, 0.3)** | K = Bangladeshi (Asian or Asian British) (n=9, 3.8) |
|  |  | Unknown/not recorded (n=61, 26.1) |
| White  (n=2,288, 85.6) | **0 = White (n=2,288, 85.6)** | A = British (White) (n=1,726, 75.4) |
|  |  | B = Irish (White) (n=2, 0.1) |
|  |  | C = Any other White background (n=35, 1.5) |
|  |  | Unknown/not recorded (n=525, 22.9) |
| Other  (n=146, 5.5) | **1 = Black Caribbean (n=4, 0.1)** | M = Caribbean (Black or Black British) (n=4, 2.7) |
|  | **2 = Black – African (n=16, 0.6)** | N = African (Black or Black British) (n=16, 11.0) |
|  | **3 = Black – Other (n=8, 0.3)** | P = Any other Black background (n=8, 5.5) |
|  | **7 = Chinese (n=1, 0.04)** | R = Chinese (other ethnic group) (n=1, 0.7) |
|  | **8 = Any other ethnic group (n=85, 3.2)** | D = White and Black Caribbean (Mixed) (n=7, 4.8) |
|  |  | E = White and Black African (Mixed) (n=2, 1.4) |
|  |  | F = White and Asian (Mixed) (n=5, 3.4) |
|  |  | G = Any other Mixed background (n=6, 4.1) |
|  |  | L = Any other Asian background (n=32, 21.9) |
|  |  | S = Any other ethnic group (n=33, 22.6) |
| *Unknown/not recorded*  *(n=7, 0.3)* |  |  |

**Table S3.** Descriptive summary of Townsend deprivation scores for the n=2,674 children included in the study cohort, for each population-weighted fifth used for analysis.

| Townsend quintile* | Median (IQR) | Range (min-max) |
| --- | --- | --- |
| Least deprived I (*n=416*) | -3.8 (-4.2, -3.5) | -5.7 to -3.1 |
| II (*n=456*) | -2.4 (-2.7, -2.1) | -3.3 to -1.6 |
| III (*n=485*) | -0.75 (-1.3, -0.3) | -1.8 to 0.6 |
| IV (*n=536*) | 1.85 (1.0, 2.5) | 0.2 to 3.5 |
| Most deprived V (*n=783*) | 5.3 (4.2, 6.8) | 3.1 to 10.6 |

*We assigned deprivation score to the patients according to the lower super output area (LSOA) of residence at the time of diagnosis, using the index preceding year of diagnosis (1997-2000 -1991 Census; 2001-2010 - 2001 Census; and 2011-2016 – 2011 Census). Townsend deprivation score was stratified into population-weighted fifths (I-V)^2^ (based on the total population of England at the time of each pertaining census)^3^

*
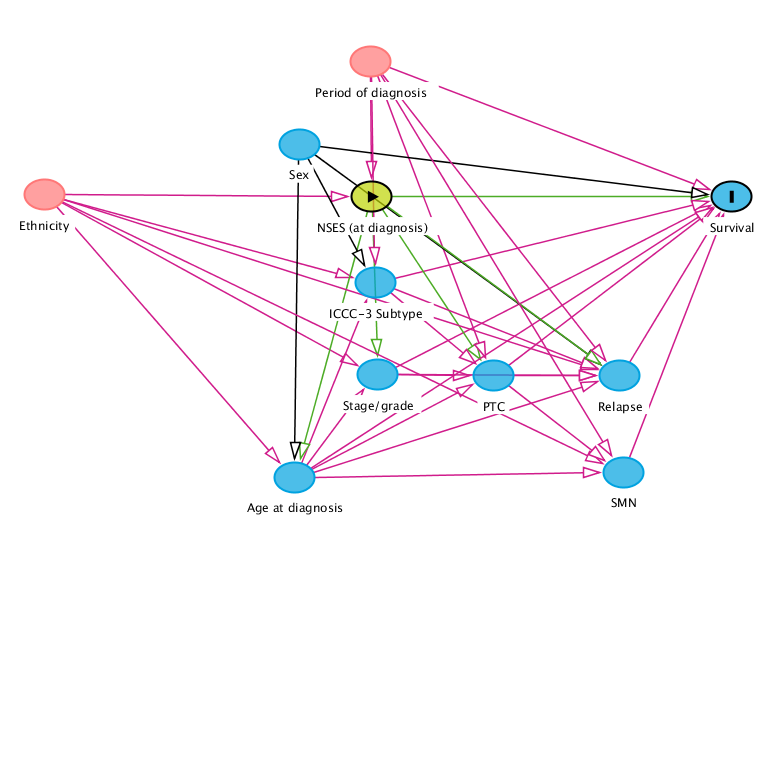
*

**Figure S1.** Directed acyclic graph (DAG) illustrating the causal effect of area-based deprivation on paediatric cancer survival

**Minimal sufficient adjustment sets**

*Minimal sufficient adjustment sets for estimating the direct effect of socio-economic deprivation on childhood cancer survival (over time):*

- Ethnic group treatment at a principal treatment centre, period of diagnosis, relapse status, sex, ICCC-3 subgroup, stage/grade, age at diagnosis

**Table S4.** Full multivariate Cox regression model details (modelling the effect of socio-economic deprivation on survival over time)

|  | **All cancers** | **Leukaemia** | **Lymphoma** | **CNS tumours** | **Other solid tumours** |
| --- | --- | --- | --- | --- | --- |
| **Period of diagnosis**  1997-2001  2002-2006  2007-2011  2012-2016 | X |  |  |  |  |
| **Period of diagnosis**  1997-2006  2007-2016 |  | X | X | X | X |
| **Ethnic group (south Asian/non south Asian)** | X | X | X | X | X |
| **ICCC-3 subgroup** | X | X | X | *Model stratified by ICCC-3 subgroup* | X |
| **Stage/grade** | X | X | X | X | X |
| **Sex** | X | X | X | X | X |
| **Age at diagnosis**^1^ | X | X | X | X | X |
| **Received treatment at a principal treatment centre (yes/no)**^2^ | X |  |  | X | X |
| **Relapse status**^1^ | X | X | X | X | X |

^1^Age and relapse status included as a time varying coefficient

**^2^** Model for leukaemia and lymphoma does not include level of treatment at principal treatment centre as care was standardised across study period. 97.5% received all treatment for leukaemia at PTC and 94.7% for lymphomas.

|  | | | Period of diagnosis | | | |
| --- | --- | --- | --- | --- | --- | --- |
|  |  |  | **1997-2006** | | **2007-2016** | |
| *ICCC-3 Diagnostic subgroup* | | ***Ethnic group*** |  |  |  |  |
| ALL | Non-south Asian | | 85.1 (80.6-88.6) | | 88.8 (84.6-91.8) | |
|  | South Asian | | 80.8 (59.8-91.5) | | 93.8 (0.77-98.4) | |
| NHL | Non-south Asian | | 85.1 (74.0-91.7) | | 96.7 (87.5-99.2) | |
|  | South Asian | | 50.0 (18.4-75.3) | | 62.5 (22.9-86.1) | |
| Astrocytoma | Non-south Asian | | 82.1 (74.1-87.8) | | 88.5 (82.2 (82.2-92.7) | |
|  | South Asian | | 71.4 (25.8-92.0) | | 87.5 (58.6-96.7) | |
| Nervous system tumours | Non-south Asian | | 58.3 (46.1-68.7) | | 70.9 (60.9-78.8) | |
|  | South Asian | | 40.0 (5.2-75.3) | | 70.0 (32.9-89.2) | |
| Bone tumours | Non-south Asian | | 52.9 (38.5-65.5) | | 70.1 (57.2-80.6) | |
|  | South Asian | | 75.0 (12.8-96.1) | | 100 (-)* | |
| Soft tissue sarcoma | Non-south Asian | | 57.9 (46.0-68.1) | | 68.4 (57.6-77.0) | |
|  | South Asian | | 100 (-)* | | 60.0 (12.6-88.2) | |
| Germ cell tumours | Non-south Asian | | 93.3 (80.7-97.8) | | 94.8 (80.8-98.7) | |
|  | South Asian | | 50.0 (0.06-91.0) | | 100 (-)* | |
| Other | Non-south Asian | | 86.7 (75.1-93.1) | | 92.2 (80.8-98.7) | |
|  | South Asian | | 90.9 (50.8-98.7) | | 86.7 (56.4-96.5) | |

**Table S5a.** Overall five-year crude survival estimates (%) for *n*=2,674 children diagnosed with cancer aged 0-14 years in Yorkshire between 1997 and 2016, by **ethnic group and 10-year period of diagnosis.**

*no deaths recorded

† Diagnoses were categorised according to the International Classification of Childhood Cancer, third edition (ICCC-3)^5^

*For those with multiple tumours (*n=*40), only first recorded tumour was included in the calculation of five-year survival rates.

¥ Townsend deprivation scores^2^ used as a measure of area-level deprivation; assigned to English population-weighted fifths for analysis where I = least deprived and V = most deprived.

**Table S5b.** Overall five-year crude survival estimates (%) for *n*=2,674 children diagnosed with cancer aged 0-14 years in Yorkshire between 1997 and 2016, by **area-based deprivation and 10-year period of diagnosis**

|  | | | Period of diagnosis | | | |
| --- | --- | --- | --- | --- | --- | --- |
|  |  |  | **1997-2006** | | **2007-2016** | |
| *ICCC-3 Diagnostic group* | *Deprivation*¥ | |  |  |  |  |
| ALL | | I | 93.1 (82.7-97.4) | | 86.8 (72.9-93.9) | |
|  | | II | 89.2 (78.7-94.7) | | 85.7 (73.5-92.6) | |
|  | | III | 81.5 (68.3-89.6) | | 88.3 (78.7-93.7) | |
|  | | IV | 78.7 (66.2-87.0) | | 90.9 (80.9-95.8) | |
|  | | V | 82.3 (73.1-88.6) | | 92.1 (84.2-96.2) | |
| NHL | | I | 83.3 (48.2-95.6) | | 90.0 (47.3-98.5) | |
|  | | II | - | | - | |
|  | | III | 80.0 (50.0-93.1) | | - | |
|  | | IV | 90.9 (50.8-98.7) | | 81.8 (44.7-95.1) | |
|  | | V | 65.4 (44.0-80.3) | | 89.5 (64.1-97.3) | |
| Astrocytoma | | I | 94.1 (65.0-99.2) | | 87.0 (64.8-95.6) | |
|  | | II | 80.0 (55.1-92.0) | | 93.6 (76.6-98.4) | |
|  | | III | 86.4 (63.4-95.4) | | 93.3 (75.9-98.3) | |
|  | | IV | 73.1 (51.7-86.2) | | 84.4 (66.5-93.2) | |
|  | | V | 80.0 (65.1-89.1) | | 85.4 (71.8-92.8) | |

- No deaths recorded

† Diagnoses were categorised according to the International Classification of Childhood Cancer, third edition (ICCC-3)^5^

*For those with multiple tumours (*n=*40), only first recorded tumour was included in the calculation of five-year survival rates.

¥ Townsend deprivation scores^2^ used as a measure of area-level deprivation; assigned to English population-weighted fifths for analysis where I = least deprived and V = most deprived.

**Table S6.** Hazard ratios (HRs) and 95% confidence intervals (CIs) from Cox regression models presenting the association between **increasing area-based deprivation***and **risk of death** in children (aged 0-14 years) with a diagnosis of acute lymphoblastic leukaemia (ALL) or high/low grade central nervous system tumours – resident in Yorkshire & the Humber between 1997 and 2016.

|  | | | | Year of diagnosis | | | | | | |  |
| --- | --- | --- | --- | --- | --- | --- | --- | --- | --- | --- | --- |
|  | **1997-2001** | | **2002-2006** | | | **2007-2011** | | **2012-2016** | | |  |
| *ICCC-3 Diagnostic group* | HR≠ | 95% CI | HR≠ | | 95% CI | HR≠ | 95% CI | HR≠ | | 95% CI |  |
| Ia. ALL | 1.17 | 1.07-1.28 | 0.93 | | 0.84-1.04 | 0.91 | 0.79-1.05 | 0.97 | 0.86-1.09 | |  |
| Low grade CNS¥ | 1.05 | 0.93-1.20 | 0.95 | | 0.78-1.17 | 1.09 | 0.94-1.26 | 0.93 | 0.76-1.15 | |  |
| High grade CNS¥ | 1.02 | 0.93-1.13 | 1.05 | | 0.96-1.16 | 1.05 | 0.96-1.14 | 0.96 | 0.88-1.06 | |  |

* Townsend deprivation scores^2^ used as a measure of area-level deprivation based on validated postcode at diagnosis. Hazard ratios present the increase in expected mortality risk with one unit increase in Townsend score, where increasing Townsend score is indicative of greater material deprivation. Hazard ratios are presented at each time period of diagnosis – to allow us to look at changes in trends over time.

≠ All HRs are mutually adjusted for confounding based on the minimal sufficient adjustment set for estimating the direct area-based deprivation on survival outcome over time, accounting for prognostic factors and patient case-mix (See Table S4 for full model details).

¥ CNS tumours were categorised according to WHO grade (low grade I-II/high grade III-VI).

**Table S7.** Hazard ratios (HRs) and 95% confidence intervals (CIs) from Cox regression models presenting the association between **increasing area-based deprivation***and **risk of death** in children (aged 0-14 years) with a cancer diagnosis – resident in Yorkshire & the Humber between 1997 and 2016. Excluding individuals (n=174) where ethnicity was determined using Onomap naming algorithm^13, 24^.

|  | Year of diagnosis | | | | | | | | |
| --- | --- | --- | --- | --- | --- | --- | --- | --- | --- |
|  | **1997-2001** | | **2002-2006** | | **2007-2011** | | **2012-2016** | | |
| *ICCC-3 Diagnostic group* | HR≠ | 95% CI | HR≠ | 95% CI | HR≠ | 95% CI | HR≠ | | 95% CI |
| All cancers combined | 1.05 | 1.01-1.10 | 1.00 | 0.95-1.06 | 1.02 | 0.97-1.07 | 1.00 0.95-1.06 | | |
| I. Leukaemia | 1.11 | 1.03-1.20 | 0.97 | 0.88-1.07 | 0.94 | 0.85-1.05 | 1.04 | 0.94-1.16 | |
| II. Lymphoma | 1.04 (0.91-1.20) | | | | 0.98 (0.78-1.23) | | | | |
| III. CNS tumours | 1.04 | 0.96-1.12 | 1.06 | 0.97-1.16 | 1.08 | 1.0 -1.16 | 0.95 | 0.87-1.04 | |
| IV-XII. Other solid tumours | 1.04 | 0.96-1.13 | 0.98 | 0.88-1.09 | 1.01 | 0.94-1.10 | 1.07 | 0.98-1.17 | |

* Townsend deprivation scores^2^ used as a measure of area-level deprivation based on validated postcode at diagnosis. Hazard ratios present the increase in expected mortality risk with one unit increase in Townsend score, where increasing Townsend score is indicative of greater material deprivation. Hazard ratios are presented at each time period of diagnosis – to allow us to look at changes in trends over time.

≠ All HRs are mutually adjusted for confounding based on the minimal sufficient adjustment set for estimating the direct area-based deprivation on survival outcome over time, accounting for prognostic factors and patient case-mix (See Table S4 for full model details).

¥ CNS tumours were categorised according to WHO grade (low grade I-II/high grade III-VI).

**Figure S2.** Kaplan–Meier survival estimates of children (0-14 years) diagnosed with lymphoma in Yorkshire between 1997 and 2016 by ethnic group (non-south Asian and south Asian) and International Classification of Childhood Cancer (third edition) diagnostic sub-group. *Abbreviations*; *NHL* = non-Hodgkin lymphoma. ‘Other’ subgroup comprises children diagnosed with miscellaneous lymphoreticular neoplasms.

**References**

1. Executive NHS. Collection of ethnic group data for admitted patients (EL/94/77). *Executive NHS*. Leeds, 1994.
2. Norman P. Identifying Change Over Time in Small Area Socio-Economic Deprivation. Applied Spatial Analysis and Policy. 2009;3(2-3):107-138.
3. Norman, Paul; “Area characteristics: Great Britain 1971 to 2011”, Mendeley Data, V1. 2017; doi: 10.17632/389scnndjy.1
4. Steliarova-Foucher E, Stiller C, Lacour B, Kaatsch P. International Classification of Childhood Cancer, third edition. Cancer. 2005;103(7):1457-1467.
